# Widespread Multidrug Resistance of *Arcobacter butzleri* Isolated from Clinical and Food Sources in Central Italy

**DOI:** 10.3390/antibiotics12081292

**Published:** 2023-08-05

**Authors:** Claudia Gabucci, Giulia Baldelli, Giulia Amagliani, Giuditta Fiorella Schiavano, David Savelli, Ilaria Russo, Stefania Di Lullo, Giuliana Blasi, Maira Napoleoni, Francesca Leoni, Sara Primavilla, Francesca Romana Massacci, Giuliano Garofolo, Annalisa Petruzzelli

**Affiliations:** 1 Istituto Zooprofilattico Sperimentale dell’Umbria e delle Marche “Togo Rosati”, 06126 Perugia, Italy; c.gabucci@izsum.it (C.G.); d.savelli@izsum.it (D.S.); s.dilullo@izsum.it (S.D.L.); g.blasi@izsum.it (G.B.); m.napoleoni@izsum.it (M.N.); f.leoni@izsum.it (F.L.); s.primavilla@izsum.it (S.P.); fr.massacci@izsum.it (F.R.M.); a.petruzzelli@izsum.it (A.P.); 2 Department of Biomolecular Sciences, University of Urbino Carlo Bo, 61029 Urbino, Italy; giulia.baldelli@uniurb.it (G.B.); ilariarusso5@gmail.com (I.R.); 3 Department of Humanities, University of Urbino Carlo Bo, 61029 Urbino, Italy; giuditta.schiavano@uniurb.it; 4 Istituto Zooprofilattico Sperimentale dell’Abruzzo e del Molise “G. Caporale”, 64100 Teramo, Italy; g.garofolo@izs.it

**Keywords:** *Arcobacter butzleri*, multidrug resistance (MDR), disc diffusion method, sushi, fresh vegetables, chicken breast, human arcobacteriosis

## Abstract

The *Arcobacter* genus comprises a group of bacteria widely distributed in different habitats that can be spread throughout the food chain. Fluoroquinolones and aminoglycosides represent the most common antimicrobial agents used for the treatment of *Arcobacter* infections. However, the increasing trend of the antimicrobial resistance of this pathogen leads to treatment failures. Moreover, the test implementation and interpretation are hindered by the lack of reference protocols and standard interpretive criteria. The purpose of our study was to assess the antibiotic resistance pattern of 17 *A. butzleri* strains isolated in Central Italy from fresh vegetables, sushi, chicken breast, and clinical human samples to provide new and updated information about the antimicrobial resistance epidemiology of this species. Antimicrobial susceptibility testing was carried out by the European Committee on Antimicrobial Susceptibility Testing (EUCAST)’s disc diffusion method. All the strains were multidrug resistant, with 100% resistance to tetracyclines and cefotaxime (third generation cephalosporins). Some differences were noticed among the strains, according to the isolation source (clinical isolates, food of animal origin, or fresh vegetables), with a higher sensitivity to streptomycin detected only in the strains isolated from fresh vegetables. Our data, together with other epidemiological information at the national or European Union (EU) level, may contribute to developing homogeneous breakpoints. However, the high prevalence of resistance to a wide range of antimicrobial classes makes this microorganism a threat to human health and suggests that its monitoring should be considered by authorities designated for food safety.

## 1. Introduction

The *Arcobacter* genus comprises a group of bacteria widely distributed in different habitats that can be spread throughout the food chain. Twenty-nine species have been recognized as belonging to the *Arcobacter* genus [1], but this number is dramatically increasing, with different potentially novel species described and detected in recent years in urban sewage, animals, and coastal waters [2,3,4,5,6]. 

Among the different species of *Arcobacter* genus, *A. butzleri*, together with *A. cryaerophilus*, *A. skirrowii*, and *A. thereius*, are considered human pathogens, and recent progress and outcomes on their pathogenicity, such as the in silico analysis by Martins et al. [7], has provided a better understanding of the resistance and virulence mechanisms. Currently, there is actually neither an Italian nor an EU regulatory requirement regarding the *Arcobacter* contamination of foods, but the designation for *A. butzleri* and *A. cryaerophilus* by the International Commission on Microbiological Specifications for Foods (ICMSF) as moderate risk for human health suggests their importance as potential foodborne pathogens [8].

Several different niches have been recognized as inhabited by *A. butzleri*, like water and raw and ready-to-eat (RTE) foods, including food products of animal origin, such as milk, cheese, seafood, and fish [9], thus highlighting that different food matrices could be the route for the spreading of this bacterium. A small number of studies about *Arcobacter* contamination in vegetables are available, but recent data revealed the presence of *Arcobacter* spp. in 20% of lettuce and 9% of rocket salad samples in Southern Italy [10], 4.4% of leafy green vegetables in Korea [11], 13.73% of vegetable samples collected from retail shops or local vendors in India [12], and 17% of fresh vegetables from Spain [13]. Several outbreaks of human gastroenteritis associated with *Arcobacter* have been linked to the consumption of contaminated fresh vegetables, which could act as an important source of infection [14,15]. Moreover, the contamination of products of animal origin has been reported more frequently in the last two decades, especially in poultry meat and meat products.

Additionally, various species of *Arcobacter* have been isolated from fish and shellfish, considered to be natural reservoir for this pathogen [16,17,18]. 

Similarly, the infectious dose has not yet been well established, and the available epidemiological data about arcobacteriosis seem to suggest a lower incidence than other foodborne diseases. This could also probably be due to the lack of routinely performed monitoring of this bacteria in clinical samples and to the absence of a standardized accurate analytical method for its detection and characterization [19], leading sometimes to misidentification with *Campylobacter* spp. Indeed, a study in South Africa showed *A. butzleri* to be the third most prevalent species in human feces, while in Belgium and France it was reported to be the fourth most prevalent species [20,21,22].

*Arcobacter* spp. has been associated with enteritis, bacteremia, endocarditis, gastroenteritis, and peritonitis [23]; it has been detected in the blood samples of patients with clinical conditions like liver cirrhosis and appendicitis [24,25], as well as from healthy humans [26]. The enteritis caused by *Arcobacter* is an acute diarrhea lasting for 3–15 days, sometimes becoming persistent or recurrent for more than two weeks or even as long as two months [27]. The condition is often accompanied by abdominal pain and nausea, and some patients also experience fever, chills, vomiting, and weakness. With a prevalence of 8%, *A. butzleri* was found to be the etiological agent of traveler’s diarrhea acquired by U.S. and European travelers to Guatemala, Mexico, and India [28,29].

Immunocompromised patients are of particular concern [30], since they may undergo recurrent infection [31]. Lastly, the possible involvement of some strains of *A. butzleri* in extra-intestinal illnesses has been shown, and experiments on in vivo models confirmed this hypothesis [32]. 

Besides the vast arsenal of potential virulence factors identified by Isidro et al. [33] through Whole-Genome Sequencing, members of the *Arcobacter* genus display characteristics that, in particular conditions, make them persistent and potentially hazardous for some categories of individuals, such as their biofilm formation capacity [34,35,36], their survival ability on different surfaces [37,38] even after sanitization and disinfection [39,40,41], and their biocide tolerance [42]. For these reasons, this genus is of clinical and veterinary importance and must be regarded as an emerging risk for human health.

Even though the symptoms of arcobacteriosis are usually self-limiting, their severity and time protraction may require antibiotic therapy. Fluoroquinolones and aminoglycosides represent the most common antimicrobial agents for the treatment of *Arcobacter* infections because they were found to be more effective compared to other antibiotics [43]. However, the available reports suggest that there is an increasing trend in antimicrobial resistance (AMR) of this emerging foodborne pathogen, leading to treatment failures with commonly used antimicrobials [44]. Indeed, different studies have confirmed the antibiotic resistance capacity of several strains of *A. butzleri* detected in clinical, water, and food specimens [33,45,46,47,48,49,50]. 

Until now, limited data about the antibiotic resistance of these strains have been available. For these reasons, this work aims to assess the antibiotic resistance pattern of 17 strains isolated in Central Italy from food matrices, such as fresh vegetables (endive, escarole, and radicchio), RTE fish products (sushi), chicken breast, and clinical samples (human feces), to provide new and updated information about the AMR features of this foodborne pathogenic species.

## 2. Results

### 2.1. Molecular Identification of Arcobacter Species

Multiplex PCR identification of the *Arcobacter* spp. strains of food and clinical origin provided amplification products referable to *A. butzleri* (372 bp) for all the samples (Table 1).

### 2.2. Antimicrobial Susceptibility Testing

The AMR was widely diffused among the tested strains (Table 2). 

**Figure 1:**
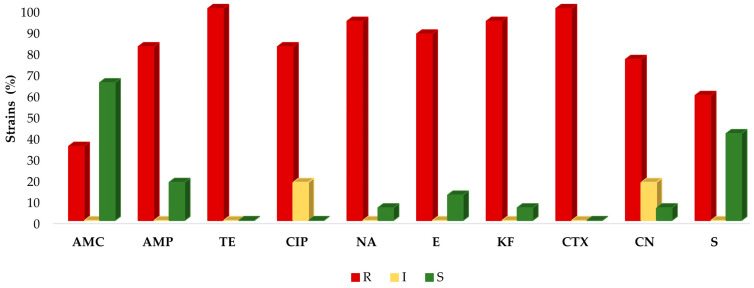
Prevalence of resistance to antimicrobials of all *A. butzleri* strains (n = 17). Resistant (red), intermediate resistant (yellow), or sensitive (green) strains to the antimicrobials tested. AMC: amoxicillin–clavulanic acid (30 μg); AMP: ampicillin (10 μg); TE: tetracycline (30 μg); CIP: ciprofloxacin (5 μg); E: erythromycin (15 μg); KF: cefalotin (30 μg); CTX: cefotaxime (30 μg); CN: gentamicin (10 μg); NA: nalidixic acid (30 μg); S: streptomycin (10 μg).

All the samples were resistant to tetracyclines and almost all to cephalosporins, except for a strain isolated from chicken breast (37809/1), which was sensitive to cefalotin. In the fluoroquinolones class, three strains (17.6%) showed intermediate resistance to ciprofloxacin, whereas the remaining were resistant; all strains but one (37809/1) (94%) were resistant to nalidixic acid. In contrast to the abovementioned results, 59% of strains were resistant to streptomycin and only 35% to amoxicillin–clavulanic acid (Figure 1). The prevalence of the resistant strains was significantly different (*p* < 0.001) among the various antibiotics tested.

**Figure 2:**
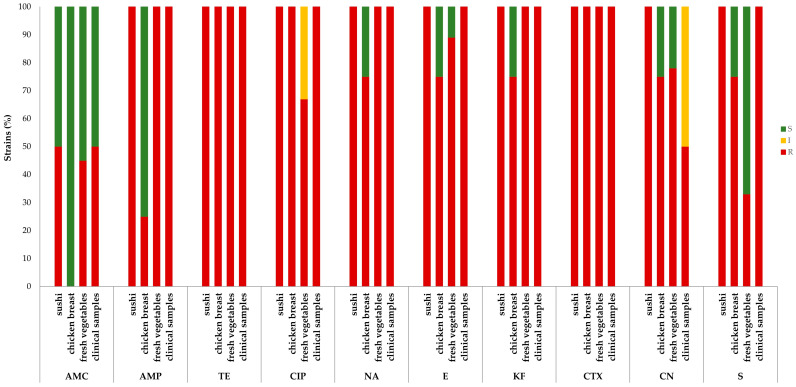
Resistance patterns of the tested strains (n=17) according to their origin. Resistant (red), intermediate resistant (yellow), or sensitive (green) strains to the antimicrobials tested. AMC: amoxicillin–clavulanic acid (30 μg); AMP: ampicillin (10 μg); TE: tetracycline (30 μg); CIP: ciprofloxacin (5 μg); E: erythromycin (15) μg; KF: cefalotin (30μg); CTX: cefotaxime (30 μg); CN: gentamicin (10 μg); NA: nalidixic acid (30 μg); S: streptomycin (10 μg).

Resistance to all antibiotics was found in one sushi-derived strain, while the other was sensitive to amoxicillin–clavulanic acid. In contrast, the strains isolated from chicken breast were all sensitive to the penicillins, except for one strain resistant to ampicillin (36981/2). Moreover, one strain (37809/1) was also sensitive to all aminoglycosides, macrolides, nalidixic acid, and cefalotin. As for the strains obtained from fresh vegetables, heterogeneous patterns were recorded: in the penicillin class, 45% of strains were resistant to amoxicillin–clavulanic acid and 100% to ampicillin. In the aminoglycoside group, 33% were resistant to streptomycin and 78% to gentamicin, with the remaining 22% showing intermediate resistance. In the fluoroquinolone class, 100% resistance to nalidixic acid and 33% intermediate and 67% full resistance to ciprofloxacin were observed. Moreover, 89% of the isolates were resistant to erythromycin, while 100% were resistant to tetracyclines and cephalosporins. One of the clinical strains (9291368) was resistant to all the antibiotic classes used in this study, while the other (8722325) was susceptible to amoxicillin–clavulanic acid and intermediate resistant to gentamicin (Figure 2). The prevalence of the resistant strains was significantly different (*p* < 0.001) according to the origin of isolation.

According to the European Centre for Disease Prevention and Control (ECDC)’s definition provided by Magiorakos et al. [51], all the *A. butzleri* strains tested were multidrug resistant (MDR).

**Figure 3:**
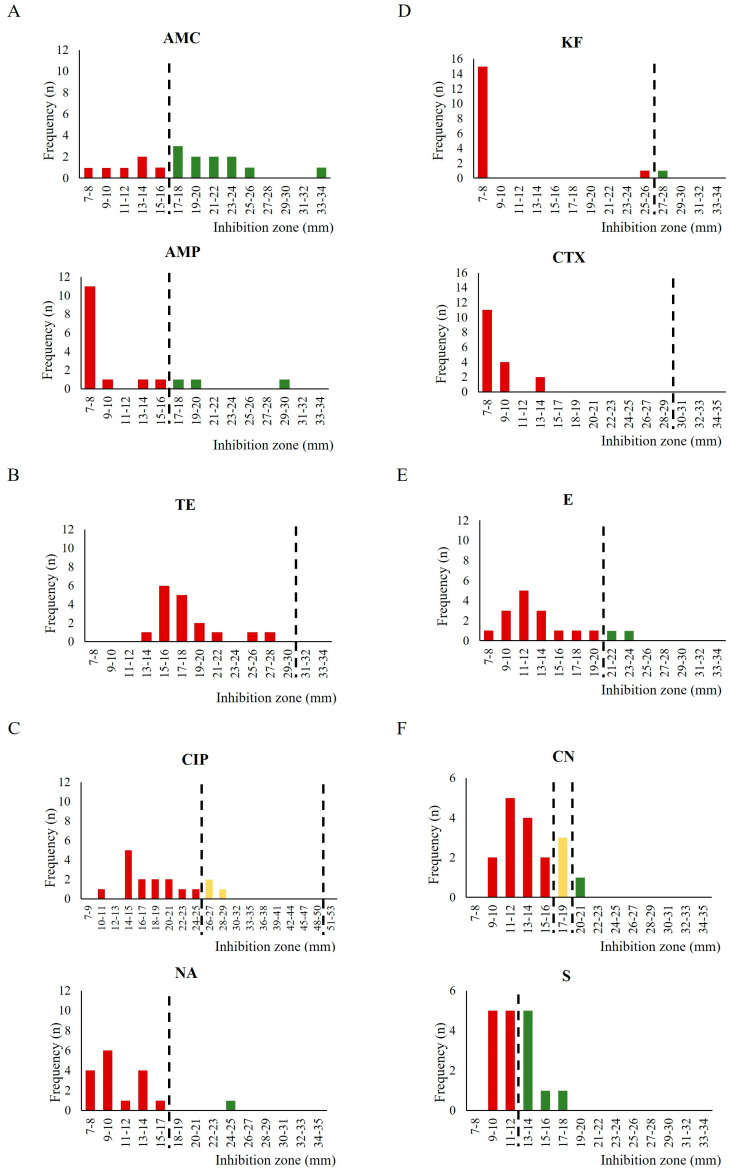
Distribution of the inhibition zone diameter in the different classes of antimicrobials obtained through the AST on the *A. butzleri* strains investigated (n = 17). The dotted lines represent breakpoints according to EUCAST [52] or BSAC [53]. Only for CIP and CN, two dotted lines are reported to indicate the intermediate resistance and the resistance cutoff values. Resistant (red), intermediate resistant (yellow), or sensitive (green) strains to the antimicrobials tested. (**A**) Penicillins: AMC: amoxicillin–clavulanic acid (30 μg) and AMP: ampicillin (10 μg); (**B**) Tetracyclines: TE: tetracycline (30 μg); (**C**) Floroquinolones: CIP: ciprofloxacin (5 μg) and NA: nalidixic acid (30 μg); (**D**) Cephalosporins: KF: cefalotin (30 μg) and CTX: cefotaxime (30 μg); (**E**) Macrolides: E: erythromycin (15 μg); (**F**) Aminoglycosides: CN: gentamicin (10 μg) and S: streptomycin (10 μg).

The inhibition zone diameter (in mm) determined by the different antibiotics used in this study at their respective concentrations were investigated more closely (Figure 3). The dimensions of the inhibition zones were widely distributed for amoxicillin–clavulanic acid (7–34 mm); in other cases, such as for streptomycin, the values were closer to the cutoff (9–18 mm), whereas for the other antimicrobials, very similar values among the strains were observed, as recorded for cefalotin (7–8 mm). 

## 3. Discussion

*Arcobacter* spp., and particularly *A. butzleri*, have gained increasing clinical significance in recent years. Due to their capacity to colonize several ecological niches, association with water reservoirs [18], and their presence in the intestinal tract and feces of healthy as well as diseased animals [54], contamination of food matrices of different categories by *Arcobacter* species may occur with a certain probability, and indeed it has been described worldwide [9,44]. Human infection occurs mainly through the ingestion of contaminated products, and fecal contamination of foods during various stages of production has been considered as the main route of contamination [55]. New consumers’ habits and trends, moving in the direction of raw or undercooked products (i.e., sushi), along with the large distribution in the food chain in industrialized countries are additional risk factors. Moreover, persistence in form of biofilm in the environments of the food production chain [34,35,36] and the resistance to disinfectants and sanitizing agents [41] have been described. All these features make *A. butzleri* a recognized human pathogen posing a potential threat to consumers’ health, with particular concern to immunocompromised people. Although the illness can be self-limited, clinical management of arcobacteriosis requires antimicrobial therapy with β-lactams, fluoroquinolones, and macrolides [56]. 

However, evidence of the AMR and MDR *Arcobacter* strains, often with high prevalence, has been reported [9]. The resistance mechanisms of *Arcobacter* to antibiotics were mainly chromosomal in nature [56]. Also, Isidro et al. elucidated the genetic bases of the AMR of *A. butzleri* and reported that this species harbors a large repertoire of efflux-pump-related genes and other antibiotic resistance determinants [33]. 

Concerning epidemiological data, the most recent studies indicated that *Arcobacter* strains frequently display high resistance rates for various antibiotic classes, including those agents currently in use for arcobacteriosis treatment. MDR rates, ranging from 20 to 93.8%, have been reported in the isolates from animals, humans, food products, and the environment [9,33,57,58,59,60,61,62].

Sciortino et al. found 100% and 89.2% *A. butzleri* resistance to the penicillin class of antimicrobials, ampicillin (AMP, 10 μg) and amoxicillin/clavulanic acid (AMC, 30 μg), respectively [46]. Similar results were shown by other authors, concerning the prevalence of resistance against ampicillin [49,63]. Our results are completely in line with those obtained by Silha and colleagues [61], who found 86.2% and 33.7% of the 80 strains isolated from poultry meat, water, and clinical sources to be resistant to ampicillin and amoxicillin/clavulanic acid, respectively.

Furthermore, Vicente-Martins, Oleastro, Domingues, and Ferreira [62] found 95.4% *A. butzleri* resistance to tetracycline (TE, 30 μg) among 65 strains isolated from retail food in Portugal; additionally, the resistance to tetracycline among all the tested strains was found in two studies [46,49], and our results confirmed these data. Also, for the fluoroquinolones, quite high percentages of resistant *A. butzleri* have been reported. From 27.7% to 41% of isolated strains in different studies were resistant to ciprofloxacin (CIP, 5 μg) [33,62], and from 93.8 to 100% were not susceptible to nalidixic acid [33,46,49,61,62,63]. These results agree with those obtained in our study for nalidixic acid, for which a 94% of resistance was assessed but not for ciprofloxacin; for this antimicrobial, in fact, we obtained 100% resistance or intermediate resistance. 

Furthermore, literature data are available for the macrolide class of antimicrobials, and only two studies assessed a high resistance prevalence in *A. butzleri* strains [49,63]. Our data confirmed these results, with 88% of strains not susceptible to erythromycin (E, 15 μg). In contrast, other authors reported lower percentages of resistance against this antibiotic [46,62]. In addition, very alarming resistance rates were also described for cephalosporins, with 78.7-100% [61,64] and 98.5-100% [46,49,62] of *A. butzleri* strains shown as not sensitive to cefalotin (KF, 30μg) and cefotaxime (CTX, 30 μg), respectively. Also in this case, our data agreed with the available results, showing 94% and 100% of resistance prevalence in the tested strains. 

Lastly, we obtained discordant results for aminoglycosides antibiotics, finding a higher percentage of resistance than those reported in the literature; particularly, we measured a 94% and 59% of resistance for gentamicin (CN, 10 μg) and streptomycin (S, 10 μg), respectively, which are in contrast to the sensitivity of almost all the *A. butzleri* strains reported by other authors [46,49,61,64]. In the present study, three strains (17.6% of the totality) were panresistant (i.e., resistant to all antibiotic classes used in this study).

To date, no reference protocols and standard interpretive criteria are available for the antimicrobial susceptibility testing (AST) of *A. butzleri*, hampering a univocal and comparable evaluation of the antimicrobial susceptibility of this bacterium [56]. The assessment of the AMR of *A. butzleri* isolates faces the difficulty that no clinical breakpoints are specified for this pathogen, yet. However, interpretative criteria for *C. jejuni* susceptibility testing recommended by the EUCAST (CIP5, E15, TE30) and BSAC zone diameter breakpoints for *Enterobacteriaceae* (AMC30, AMP10, NA30, KF30, CTX30, CN10, S10) were used to analyze the results [53,65,66], as previously reported by other authors [9,49,61,62,63,64]. 

Considering the lack of *Arcobacter* specific cutoff values, the graphical representation of the distribution of the inhibition zone diameters used in this work (Figure 3) could be more useful to compare our data with other epidemiological information at the national or EU level, in order to contribute to realize an epidemiological cutoff (ECOFF) [67]. Interestingly, arbitrarily analyzing the obtained results with the epidemiological cutoffs suggested by Zautner et al. [50], a much higher percentage of susceptible strains to ciprofloxacin, tetracycline, and erythromycin could be observed. For this reason, strains that exhibit smaller inhibition zones diameter, which were also considered to be resistant according to the EUCAST or BSAC guidelines, will be interesting candidates for investigating the genetic basis of the antibiotic resistance by Whole-Genome Sequencing. Most importantly, to standardize the information on the resistance pattern of this pathogen, it will be necessary for homogeneous breakpoints to be developed, at least at the European level.

Lastly, prevention and control measures to limit the pathogen spread and the related infections should also include food acid treatment (citric and lactic acid at 1–2%), good cooking practices, effective treatment of water resources and monitoring for their contamination, maintenance of slaughter hygiene and carcass control, HACCP, and good manufacturing practice application [44].

Certainly, the increasing and alarming drug resistance scenario demands the attention of researchers to find novel and alternative therapeutic options for preventing and controlling the spread of *Arcobacter* spp. in an effective way; this would be beneficial with regard to both public health and food safety viewpoints. *A. butzleri* AMR represents, therefore, a theme of concern; in this perspective, the investigation about the possible use of polyphenols as resistance modulators in *A. butzleri* has been proposed, showing that some stilbenes can increase its sensitivity, probably acting as efflux pump inhibitors [68].

## 4. Materials and Methods

### 4.1. Bacterial Strains

*Arcobacter* spp. food strains (n. 15), collected from June to October 2022 were included in this study. Bacterial strains were isolated from sushi (12%), fresh vegetables (53%), and chicken breast (23%), according to the isolation protocol proposed by Collado et al. [69]. 

The food matrices investigated were chosen based on the epidemiological data about *Arcobacter* prevalence in such matrices [9], including food items that are commonly eaten raw without any preparation phase.

Briefly, isolation was obtained after a first enrichment step of the food sample in Arcobacter broth supplemented with 8 mg/L cefoperazone, 10 mg/L amphotericin B, and 4 mg/L teicoplanin (Thermo Scientific, Waltham, MA, USA); then, the enriched broth was inoculated by passive filtration on Tryptic Soy Agar + 5% sheep blood, and incubated at 30 °C in a microaerophilic atmosphere generated using a jar gassing system (CampyGen 2.5L, Thermo Scientific). The suspected colonies were identified based on morphological criteria (small and round, with a translucent to beige color), Gram stain (Gram-negative), and oxidase test (oxidase-positive). Moreover, the study also included the analysis of two clinical strains (12%) isolated in 2018 and in 2022 from human feces of patients with acute gastroenteritis. Both clinical strains were isolated from patients undergoing acute infection with intestinal symptoms, who sought hospital care. The patients were a male and a female, aged 59 and 69, respectively, at the time of *Arcobacter* isolation. No further information about the patients nor the clinical course of infection was available.

The *Arcobacter* strains were routinely grown at 30 °C on Mueller Hinton (MH) agar plates (Thermo Scientific) supplemented with 5% defibrinated horse blood (Liofilchem, Roseto degli Abruzzi, Italy). All incubations were at 30 °C under microaerophilic conditions.

### 4.2. Multiplex PCR-Based Species Identification

All the strains were identified at the species level by multiplex PCR, with primers Cpn60-F (GCA CAT TCT ATT TTC AAA GAA GGG) and Cpn60-R (GAA TGG GTT ATT AAA CTC TGC) for *A. butzleri*; GyrAcry-F (AGT TCT GAA GCA ATA GAT TTA ATG G) and GyrAcry-R (CTG CAA TTC CTT CGA TTT GC) for *A. cryaerophilus*; Skirr-F (GGC GAT TTA CTG GAA CAC A) and Skirr-R (CGT ATT CAC CGT AGC ATA GC) for *A. skirrowii*. *A. butzleri* DSM 8739, *A. cryaerophilus* DSM 7289, and *A. skirrowii* DSM 7302 were used as positive controls [70].

### 4.3. Antibiotic Susceptibility Tests

The AST of the isolated strains was performed against ten antibiotics through the European Committee on Antimicrobial Susceptibility Testing (EUCAST)’s disc diffusion method [71]. The strains were tested against: amoxicillin–clavulanic acid (AMC, 30 μg; AMC30), ampicillin (AMP, 10 μg; AMP10), cefalotin (KF 30 μg; KF30), cefotaxime (CTX, 30 μg; CTX30), ciprofloxacin (CIP, 5 μg; CIP5), erythromycin (E, 15 μg; E15), gentamicin (CN, 10 μg; CN10), nalidixic acid (NA 30 μg; NA30), streptomycin (S, 10 μg; S10), and tetracycline (TE, 30 μg; TE30) (Oxoid, UK). The isolates were subcultured on MH agar supplemented with 5% defibrinated horse blood (Thermo Scientific) with incubation at 30 °C for 48 h under microaerophilic conditions. A 0.5 McFarland bacterial suspension was prepared in BHI broth, spread on MHF, and the antibiotic test discs were positioned. After incubation for 48 h at 30 °C in a microaerophilic atmosphere, the diameters of the inhibition zones were measured. The bacterial isolates with insufficient growth after 48 h were re-incubated, and the inhibition zone was measured to the closest millimeter after a total of 72 h. The experiments were performed in triplicate. The quality control strains used for resistance testing by agar diffusion were *Campylobacter jejuni* ATCC 33560 for the antibiotic test discs CIP5, E15, and TE30, and *Enterococcus faecalis* ATCC 29212 for AMP10 and AMC30.

According to other authors [1,45,46,62], since no breakpoints have been defined for *Arcobacter* spp. either by the EUCAST or the Clinical & Laboratory Standards Institute (CLSI), the strains were classified as resistant (R), susceptible (S), or intermediate (I) on the basis of EUCAST breakpoint tables for interpretation of the zone diameters [52] given for *C. jejuni* (CIP5, E15 and TE30) [52]. Moreover, the strains’ sensitivity to antibiotics other than those listed for *C. jejuni* was evaluated according to the interpretation guidelines of the British Society for Antimicrobial Chemotherapy (BSAC) Resistance Surveillance Programme [53,66] for *Enterobacterales*.

The strains resistant to at least three classes of antibiotics were classified as MDR [51].

### 4.4. Statistical Analysis

The chi-square test was used to assess the differences between the prevalence of the resistant strains to the antibiotics tested. Separate analyses were conducted on all the isolates or grouped according to their origin. *p* values lower than 0.05 were considered statistically significant. 

## 5. Conclusions

The high prevalence of AMR to a wide range of antimicrobial classes found in this study for *A. butzleri*, including a percentage of resistance to aminoglycoside antibiotics higher than that reported in previous research, should be considered an important finding, especially since aminoglycosides are the main therapeutic option for human arcobacteriosis. 

The scarce or unknown incidence of *Arcobacter* spp. infections, along with the lack of standardized methods for the detection of the pathogen and the assessment of its AMR may determine an underestimation of the potential threat to human health. Therefore, our data provide a contribution and suggest that *Arcobacter* spp. monitoring and the epidemiological surveillance of its AMR features should be considered by the authorities designated for food safety.

## Figures and Tables

**Table 1:** *Arcobacter* strains included in this study, with isolation sources.

Strain ID	Species	Isolation Source	Isolation Date
31164/3	*A. butzleri*	Sushi	July 2022
35709/3	*A. butzleri*	Sushi	August 2022
35683/1	*A. butzleri*	Chicken breast	August 2022
36981/2	*A. butzleri*	Chicken breast	August 2022
37809/1	*A. butzleri*	Chicken breast	August 2022
39884/1	*A. butzleri*	Chicken breast	August 2022
25176/2	*A. butzleri*	Fresh vegetables, curly endive	June 2022
29991/1	*A. butzleri*	Fresh vegetables, escarole	June 2022
32455/2	*A. butzleri*	Fresh vegetables, curly endive	July 2022
35638/2	*A. butzleri*	Fresh vegetables, curly endive	August 2022
40619/1	*A. butzleri*	Fresh vegetables, escarole	September 2022
43130/1	*A. butzleri*	Fresh vegetables, escarole	September 2022
43130/2	*A. butzleri*	Fresh vegetables, curly endive	September 2022
43130/3	*A. butzleri*	Fresh vegetables, radicchio	September 2022
45224/3	*A. butzleri*	Fresh vegetables, chicory	October 2022
8722325	*A. butzleri*	Clinical isolate	September 2018
9291368	*A. butzleri*	Clinical isolate	August 2022

**Table 2:** The results of the antibiotic susceptibility tests of the 17 *Arcobacter* strains to 10 different antimicrobials with the disc diffusion method (EUCAST, 2023 and BSAC, 2015).

			Penicillins		Tetracyclines	Fluoroquinolones		Macrolides	Cephalosporins		Aminoglycosides	
		Strain ID	AMC	AMP	TE	CIP	NA	E	KF	CTX	CN	S
Food matrices	Sushi	31164/3	R	R	R	R	R	R	R	R	R	R
		35709/3	S	R	R	R	R	R	R	R	R	R
	Chicken breast	35683/1	S	S	R	R	R	R	R	R	R	R
		36981/2	S	R	R	R	R	R	R	R	R	R
		37809/1	S	S	R	R	S	S	S	R	S	S
		39884/1	S	S	R	R	R	R	R	R	R	R
	Fresh vegetables	25176/2	S	R	R	R	R	R	R	R	I	S
		29991/1	R	R	R	I	R	S	R	R	R	S
		32455/2	S	R	R	I	R	R	R	R	R	S
		35638/2	R	R	R	R	R	R	R	R	R	R
		40619/1	S	R	R	R	R	R	R	R	I	S
		43130/1	R	R	R	I	R	R	R	R	R	R
		43130/2	S	R	R	R	R	R	R	R	R	S
		43130/3	R	R	R	R	R	R	R	R	R	S
		45224/3	S	R	R	R	R	R	R	R	R	R
Clinical		8722325	S	R	R	R	R	R	R	R	I	R
		9291368	R	R	R	R	R	R	R	R	R	R

Background colors are for resistant (red), intermediate (yellow), or sensitive (green) strains. AMC: amoxicillin–clavulanic acid (30 μg); AMP: ampicillin (10 μg); TE: tetracycline (30 μg); CIP: ciprofloxacin (5 μg); E: erythromycin (15 μg); KF: cefalotin (30 μg); CTX: cefotaxime (30 μg); CN: gentamicin (10 μg); NA: nalidixic acid (30 μg); S: streptomycin (10 μg).

## Data Availability

The datasets generated and/or analyzed during the current study are not publicly available but are available from the principal investigator (PI) on reasonable request.
